# Statistics and pitfalls of trend analysis in cancer research: a review focused on statistical packages

**DOI:** 10.7150/jca.43521

**Published:** 2020-03-04

**Authors:** Jie Xu, Yong Lin, Mu Yang, Lanjing Zhang

**Affiliations:** 1 Department of Infectious Disease, Shanghai Ninth People's Hospital, Shanghai Jiao Tong University School of Medicine, Shanghai, China.; 2 Rutgers Cancer Institute of New Jersey, New Brunswick, New Jersey.; 3 Department of Biostatistics, School of Public Health, Rutgers University, Piscataway, New Jersey.; 4 Department of Pathology, Shanghai First Hospital, Shangai Jiao Tong University, Shanghai, China.; 5 Department of Pathology, Princeton Medical Center, Plainsboro, New Jersey.; 6 Department of Biological Sciences, Rutgers University, Newark, New Jersey.; 7 Department of Chemical Biology, Rutgers Ernest Mario School of Pharmacy, Piscataway, NJ.

**Keywords:** statistical analysis, software, cancer, nonlinear trend, joinpoint regression, linear spline regression.

## Abstract

Trend analysis is the analysis using statistical models to estimate and predict potential trends over time, space or any independent continuous-variable. It has been widely used in epidemiology and public health, but much less so in clinical oncology and basic cancer research. Methodological imitations of the chosen statistical package also appear to result in biased or less rigorous interrogation of cancer-related data. We thus review the basic statistics of trend analysis, commonly used commands of statistical packages and the common pitfalls of conducting trend analysis. Four free and 3 commercial statistical-packages were discussed in depth, including Joinpoint, Epi info, R package, Python, SAS, Stata and SPSS. We hope that this review could serve as a practical yet concise guide for using statistical packages for trend analysis in translational and clinical oncology, and help improve the scientific rigor of trend analyses in these fields. The guide, however, may also be applied to other research fields.

## Introduction

Trend analysis has been widely used in the cancer epidemiology 1, 2. The capacity to predict future trends and inferencing past trends is one of the major advantages of trend analysis. However, the statistics of trend analysis is often inappropriately conducted or reported 3, 4. We also found low rates in reporting confidence/credibility/prediction intervals and *p* values among the trend analyses published in leading medical and oncology journals (personal data), although reporting estimated effect size and confidence/credibility/prediction intervals is highly recommended 5, 6. Inappropriate reporting and conducting of trend analysis may lead to not only less scientific rigor of the published works, but also misleading or incorrect scientific conclusions and subsequently unintended-harms to our patients. We therefore provide a practical yet concise guide on the statistics and pitfalls of trend analysis on clinical and translational oncology, with a focus on piecewise-linear models.

## Applications in translational and clinical oncology

Translational oncology is the bridge between the basic science and clinical oncology, while clinical oncology is mostly focused on clinical aspects of oncology. Through translational oncology, the breakthroughs of basic science are applied to patients (bench-side to bed-side). and the clinical inquiries lead to clinically impactful scientific discoveries (bed-side to bench-side). It in our view synergize the advances of both ends. Trend analysis, as a useful quantitative model/tool, can certainly play an important role in quantitative biology and computational oncology. We recently identified the upward use of high throughput technology in the genomic data deposited in Gene Expression Omnibus 7, in which 32.5% were human genomic data on cancer. Following cancer, the second and third popular subjects in the Gene Expression Omnibus only covered 6.1% and 4.4% of all deposited data, respectively. We thus anticipate a significant increase in research on human cancer genomics in the near future, and more application of quantitative biology methods including trend analysis.

Moreover, trend analysis has been widely used in clinical medicine, public health and cancer epidemiology 1-3, 8, 9. Relevant guidelines were published on how to best conduct trend analysis using the data of National Center for Health Statistics, while many unanswered questions remain outstanding 8. In light of the great use of trend analysis in clinical medicine, public health and cancer epidemiology, we here advocate more and better use of trend analysis in translational medicine and basic science because it will certainly transform the status quo of qualitative biology mode/models to quantitative modes/models, that are more precise and complex. For example, the piecewise linear/nonlinear models would predict a change in the association of exposures (i.e. independent variables) and the outcome (i.e. dependent variable) as the exposures reach to a data point and additional factor(s) may become associated with the outcome. Currently, linear or binary models are often, if not always, used to fit the biological mechanisms. The multifactorial and complex real world may not be well explained or fit by the rudimentary binary or (log-)linear models, while those models work in many occasions. We thus believe that application of trend analysis, particularly that of multivariable and piecewise models, may provide a quantitative, additive model of multiple factors' effects on a given outcome. The recently reported change in trend of thyroid cancer incidence 1 probably could be better modelled using piece-wise linear regression model, using the data of a rather long-study period (1974-2016) and multiple changing points 1, 10. It will thus drastically transform translational and basic biomedical sciences, and help develop more sophisticated biological models and hypotheses.

Finally, modern statistical-learning models such as machine learning, deep learning and convolutional neural network of artificial intelligence could be applied to translational medicine through trend analysis, whereas the machine learns and develop proper algorithms for modeling the trends and predict the future data points. Caution should be used when the dataset is of small size and the performance of these statistical-learning models is not compared with conventional statistical models.

## Statistical notes

Trend analysis is an analysis using statistical models to estimate and predict potential trends over time, space or any independent continuous-variable 8. Such a trend could be linear, nonlinear or absent. For linear trends, ordinary least square regression is probably the simplest and most commonly used. For possible nonliear trends, National Center for Health Statistics Guidelines recommend to use one of the 4 models, including polynomial regression, orthogonal polynomial contrasts, joinpoint regression, and restricted cubic spline regression 8. Additional models may also be used such as exponential and quadratic models. Moreover, either logistic or linear models can fit binary outcomes, while Cochran-Amitage test for trend can be used to fit ordinal category-outcomes 8.

When no clear parametric models can fit the record-level data and continuous time/space points, discrete time/space points (often start and end points) can be used for comparison 4, 8. However, comparison of 2 data-points probably should be considered as difference analysis. Bayesian models are gaining more attention in recent years 8, 11.

## Related commands in statistical packages

Many statistical packages can be used for trend analysis. We here recommend 4 free and 3 commercial statistical-packages, which are popular among statisticians and epidemiologists. Despite their sufficient functions for trend analysis, all of them like any statistical program have their own advantages and disadvantages. Therefore, the veterans, who have experiences in a statistical program, probably should continue using the one(s) they use unless the package will soon be discontinued or unavailable. The beginners should first consult with local experts and colleagues about the expertise and support of available statistical programs/packages before locking in any of them. They should then join and learn from the software/package community, which was devoted to the specific package, for trouble-shooting and learning more-advanced skills.

For piecewise linear models, the free **Joinpoint Regression Program** is probably the most user-friendly, yet reasonably functional, statistical package for linear and jointpoint trend-analysis 12, 13. It is capable to compare the trend-slopes and identify the best-fit model for the number and position(s) of the joinpoints (turning-points), by which trend slopes intercept. One noteworthy tip of using the Jointpoint Regression Program is that all data must be sorted by the time/space-point variable as the last level of sorting. The other is that it can automatically compute the secondary parameters (e.g. %, ratio, etc), their variances and their potential trends if standard-errors or both numerators and dominators provided. However, this package cannot conduct multivariate analyses and is hence only useful for descriptive analyses. One study on mortalities of hepatocellular carcinoma and liver cirrhosis is an example of such limitation 14. Neither can the Joinpoint package properly handle missing data; one must replace missing data using imputation methods or omit the time/space-point with data in the analysis. We further found its latest version (4.7.0.0, Feb. 2019) was more data-format sensitive than the prior version (4.6.0.0, April 2018), despite many added functions 15.

**Epi Info™** is another free statistical program, and can be used for both temporal and spatial linear-trend analysis.^16^ It is particularly useful for geographic visualization in maps and for data collection through web surveys. Through its advanced-statistics menu, Epi Info™ performs linear regression (REGRESS command) and supports automatic dummy variables and multiple interactions. We were also impressed with its dual syntax- and graphic-user interfaces (GUI), availability for multiple platforms (Mac®, Windows®, Android®, Iphone® and cloud computing), and sophisticated data-management function. It, however, cannot test slope parallelisms or conduct piece-wise linear regression.

The open-source, free **R package** is widely used in bioinformatics/biostatistics field. It is based on commands/syntaxes, but can be accessed using various GUI suites (e.g. RStudio). Its basic linear-regression command is *fit <- lm(y ~ x1 + x2 + x3, data=mydata), summary(fit) # show results*The “segmented” library (package) could identify change point(s) of the trends as piecewise linear regression 17. Postestimation analyses including Davies' analysis (syntax: *davies.test(fit.glm,"variable_name", k=number_of_points*) and slope tests (syntax: *slope(fit)*) will be needed to produce more detailed inferential data on the trends before and after the change points.

**Python** is an open-source (free), increasingly adopted high-level, general-purpose programming language 18. It has been widely used in artificial intelligence fields including machine learning and deep learning for its faster speed and relatively intuitive/simple grammar 19. For linear regression with Python, one could use numpy (syntax:* import numpy as np; from statsmodels.formula.api import ols; model = ols('y ~ x1 + x2', data).fit()*) or scikit-learn library (syntax: *from sklearn.linear_model import LinearRegression; model = LinearRegression(); model.fit(x, y)*)^19^, ^20^. For piecewise linear regression, one could use the command of *numpy.piecewise(), interpolate.splrep()* or *pwlf.PiecewiseLinFit()* 21. However, data cleansing with python may be challenging. Another challenge of using Python is the lack of GUI, which may be difficult to overcome for investigators who are not familiar with syntaxes.

The three popular commercial statistical-packages all can perform trend analysis. Briefly, the simple linear-regression syntaxes are *proc reg; model y=x;*in **SAS®**, *regress y x1 x2 x3* in **Stata®** and *regression /statistics coeff outs r anova ci /dependent y /method = enter x1 x2 x3* in **SPSS®**.^22^ Quadratic modelling can be performed using proc glm data=data; model y=x x*x in **SAS®**. Our experiences and others' show that these commercial packages are all sufficient for common trend analysis including piecewise linear regression, and relatively easy to use with different learning curves. However, we still recommend to consult a biostatistician about the limitations of a commercial statistical-package of your interest. Moreover, complex menus and comprehensive lists of functions of these packages may be overwhelming for beginners. Furthermore, subtle syntax modifications may have some unintended consequences. Therefore, careful review of the codes and output is highly recommended. Finally, costs and version-compatibility may be concerning to some users.

## Common pitfalls

Common errors in data management should first be prevented, such as misaligning data labels, mishandling of missing data, and errors in transforming data. Several pitfalls are common in trend analysis, and may be avoided using a checklist (**Table 1**).

We also recommend the following considerations: **1**, When a subgroup of the study population has an insufficient number of samples, data-point aggregation (pooling) may be statistically sound and can increase the sample size. However, the number of data-points for each aggregated data-point (e.g. combine 3 data-points into 1) should be as small as possible so that potential turning points could be detected. Indeed, hypothesis tests tend to be different results even if the trend/slope variances of aggregated- and record-level data are similar 8. **2**, Examination of the model fitting is critical, 11 but often overlooked 4, 8. As recommended by Woodward, residuals (error generated by a model) and influences should be checked for linear regression models 11. To our knowledge, Epi Info, R-package, Stata, SAS and SPSS can report residuals of regression models while Joinpoint only returns Statistics (*t* values). **3.** We recommend to examine the data using an internal control, which should be a variable with known increasing or decreasing trend. **4**. A simple linear regression model and a piecewise-linear model may be both valid statistically, but the former is preferred for an overall trend and the latter is for the data of long study-period or those beyond a simple linear-trend. **5**. Nonparametric models or tests are sometime the best way to examine the potential trends, and are available in R-Package, SAS, Stata and SPSS.

## Summary

This practical yet concise guide is focused on statistical packages for trend analysis in cancer research. It was intended to serve as a quick reference for trend analysis on clinical and translational oncology and a remedy for its common pitfalls, while the guide may also be applied to other fields. However, we recommend authors and reviewers to seek more professional and sophisticated instructions through biostatistical consultation, articles/guidelines 4, 8, books and educational websites when needed 22. Investigators are also recommended to read and refer to the tutorials and documentations of statistical packages, which usually provide practical guides, useful examples and pertinent theoretical frameworks. Finally, we call for research, development and publication of the guidelines on reporting trend analyses.

## Figures and Tables

**Table 1:**
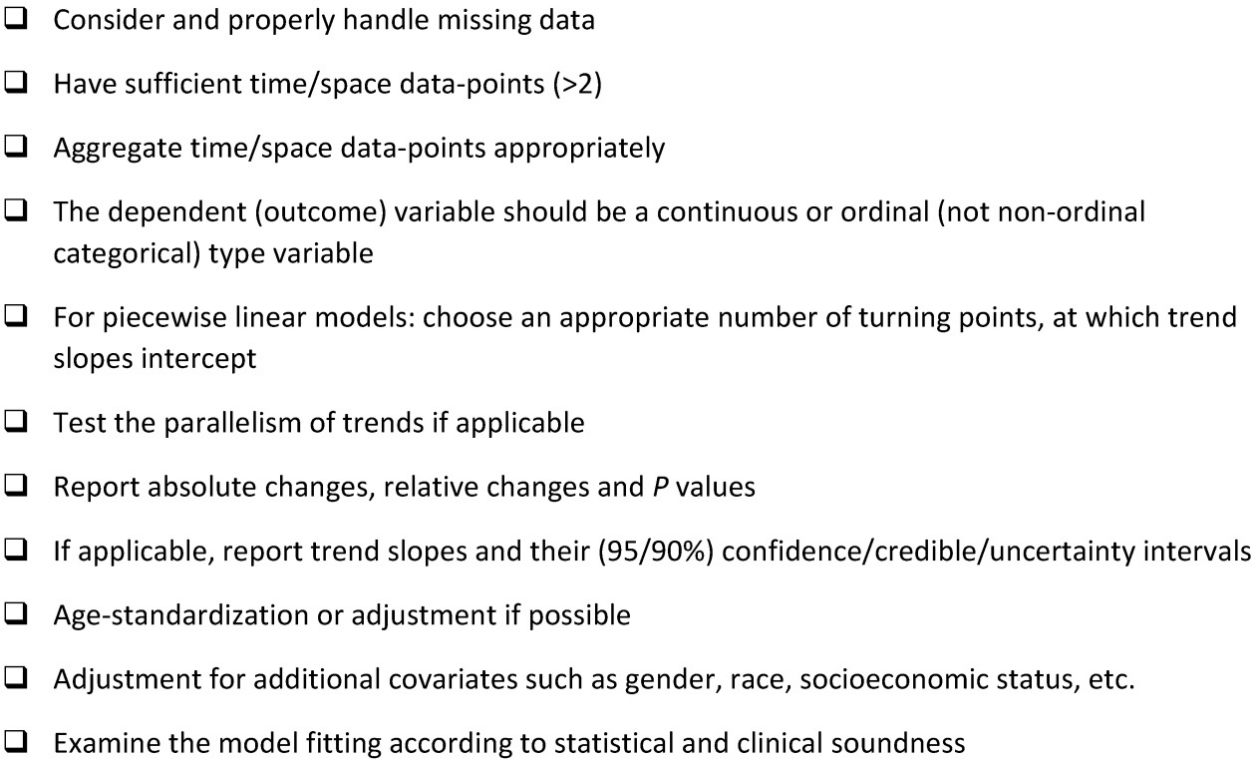
A short checklist for conducting trend analysis.
